# Fur-regulated urease contributes to the environmental adaptation of *Yersinia pseudotuberculosis*

**DOI:** 10.1128/spectrum.02756-24

**Published:** 2025-02-25

**Authors:** Junyang Wang, Peishuai Fu, Xinquan He, Yuqi Liu, Yuxin Zuo, Zhiyan Wei, Yao Wang, Yantao Yang, Changfu Li, Xihui Shen, Lingfang Zhu

**Affiliations:** 1 State Key Laboratory for Crop Stress Resistance and High-Efficiency Production, Shaanxi Key Laboratory of Agricultural and Environmental Microbiology, College of Life Sciences, Northwest A&F University, Yangling, Shaanxi, China; Shandong University, Qingdao, China

**Keywords:** urease, Fur, Mn^2+^, *Yersinia pseudotuberculosis*

## Abstract

Urease converts urea into ammonia and carbon dioxide, providing a nitrogen and carbon source for microbial growth and serving as an important mechanism for human bacterial pathogens to survive in acidic conditions, which can be regulated by many factors. As a global regulator, the ferric uptake regulator (Fur) regulates a series of genes and pathways involved in many different cellular processes and the virulence of the enteric bacterium *Yersinia pseudotuberculosis* (*Yptb*). However, whether Fur regulates the urease activity in *Yptb* was still unknown. In this study, we found that urease is positively regulated by Fur in response to manganese ions (Mn^2+^), and this regulation by Fur is mediated by specific recognition of the promoter region of urease in *Yptb*. Furthermore, urease is induced by Mn^2+^ via Fur under low nutrient conditions. Moreover, we provided evidence that urease plays an important role in acid and osmotic stress resistance, biofilm formation, and virulence of *Yptb*. Our findings provide insights into understanding the regulatory mechanism and multiple functions of urease in *Yptb*.

**IMPORTANCE:**

Urease catalyzes the breakdown of urea into ammonia and carbamate, which are widely distributed among bacterial species and play an important role as an important acid resistance system and virulence factor. In most bacterial species, urease expression is tightly regulated in response to environmental cues such as nitrogen status, pH, growth phase, substrate availability, or transcriptional regulators. In this study, we found that urease from *Yptb* is positively regulated by Fur in response to Mn^2+^ under low nutrient conditions, which functions to combat acid and osmotic stress and enhance biofilm formation, and plays a crucial role in virulence. Importantly, this is the first demonstration of a direct role for Fur and Mn^2+^ in regulating urease expression in *Yptb*. This study provides a comprehensive understanding of the regulatory mechanisms and functions of urease from *Yptb*.

## INTRODUCTION

*Yersinia pseudotuberculosis* (*Yptb*) is a Gram-negative bacterium commonly associated with foodborne illness, causing mild gastrointestinal diseases such as mesenteric lymphadenitis, appendicitis, and ileitis, as well as secondary complications such as diarrhea (1). Due to its transmission through food and water, *Yptb* often encounters acidic environments during infection, highlighting the importance of acid resistance for the survival and colonization of gastrointestinal pathogens (2). As a result, bacteria have evolved four acid resistance systems (AR1–AR4) to enhance survival (3). A functional AR3 has been reported in *Yptb*, which is crucial for bacterial survival under strong acidic conditions. Our previous study has shown that the OmpR-regulated type VI secretion system (T6SS) clusters, T6SS4, is essential for bacterial survival under acidic conditions (4). In addition, *Yptb* also uses urease to neutralize H^+^ for tolerance to weakly acidic conditions by converting urea into ammonia (5).

Bacterial urease is a metalloenzyme with an active site containing nickel ions (Ni^2+^), capable of hydrolyzing urea into ammonia (NH_3_) and carbon dioxide (CO_2_), increasing in environmental pH (6). The bacterial urease gene cluster consists of three main sections: structural (*ureABC*), auxiliary (*ureEFGD*), and regulatory genes (7). Notably, *ureC* is highly conserved among bacterial species, and its urease-active neutral region resides in the α-subunit, which is critical for function (8). As a crucial acid-resistance system and virulence factor, urease is prevalent in microorganisms such as *Helicobacter pylori*, *Staphylococcus aureus*, and *Yersinia enterocolitica* (9–11). Urease is responsible for acid resistance and contributes to the virulence of *Y. enterocolitica* by increasing the likelihood of bacterial survival during passage through the stomach (12, 13). However, the precise biological roles and molecular mechanisms of the urease from *Yptb* have not yet been fully elucidated.

In most bacterial species, urease expression is tightly regulated by substrate availability, growth stage, nitrogen status, pH, and even transcriptional regulators (14, 15). Our previous studies have highlighted that *Yptb* urease is regulated by various transcriptional regulators and environmental factors, including OmpR, CsrA, and RovM (2, 5). Among them, OmpR positively regulates the expression of urease, which enhances acid resistance (5), while CsrA negatively regulates the expression of urease (2). In *H. pylori*, the two-component system ArsRS, as well as a nickel response regulator NikR, sense the change in pH to activate urease expression under acidic conditions (16). A nickel-binding protein Mua represses urease expression when nickel levels are high, thus counterbalancing the NikR-mediated activation (17). In *Proteus mirabilis*, the histone-like nucleoid structuring protein H-NS has been observed to repress urease activity by repressing UreR, a urease transcriptional activator, in the absence of urea induction (18). In conclusion, it is evident that bacterial urease is subject to extensive regulation by a complex regulatory network.

Fur, known as the ferric uptake regulator, plays a crucial role in regulating iron homeostasis and metabolic pathways in bacteria (19). In many pathogenic bacteria like *Pseudomonas aeruginosa*, *Vibrio spp*, and *Legionella pneumophila*, Fur senses a diversity of environmental stimuli and plays a crucial role in pathogenesis through regulating the expression of iron transport systems, virulence factors, and toxins in host-bacterial interactions (19). Moreover, Fur is involved in the coordination of the oxidative and acid stress defense mechanisms within the cell. *H. pylori* has a Fur-dependent two-step acid tolerance response that enhances its capacity to survive in more acidic environments (20). In *Yptb*, Fur-regulated T6SS4 and aerobactin siderophore systems contribute to oxidative stress resistance and virulence (21–23). It is noteworthy that Fur has been shown to regulate urease expression to promote bacterial survival in *H. pylori*, *Escherichia coli*, *Helicobacter hepaticus*, and *Klebsiella pneumonia* (24–28). Whether the specific link between *Yptb* urease and Fur exists is worth exploring.

In this study, we found that Fur activates urease expression by binding directly to its promoters in *Yptb* YPIII, and further studies showed that Fur-mediated expression of urease responds to Mn^2+^ under low nutrient conditions. Urease contributes to stress resistance, enhancing the survival of *Yptb* in acidic and hypertonic environments. Furthermore, urease affects the ability of *Yptb* to form biofilms and to colonize mice, thereby increasing its pathogenicity. This study provides new insights into the regulation of urease via the Mn^2+^-dependent transcriptional regulator Fur and deepens our understanding of the regulatory mechanisms and functions of urease from *Yptb*.

## RESULTS

### Fur positively regulates urease expression in *Yptb*

**Fig 1:**
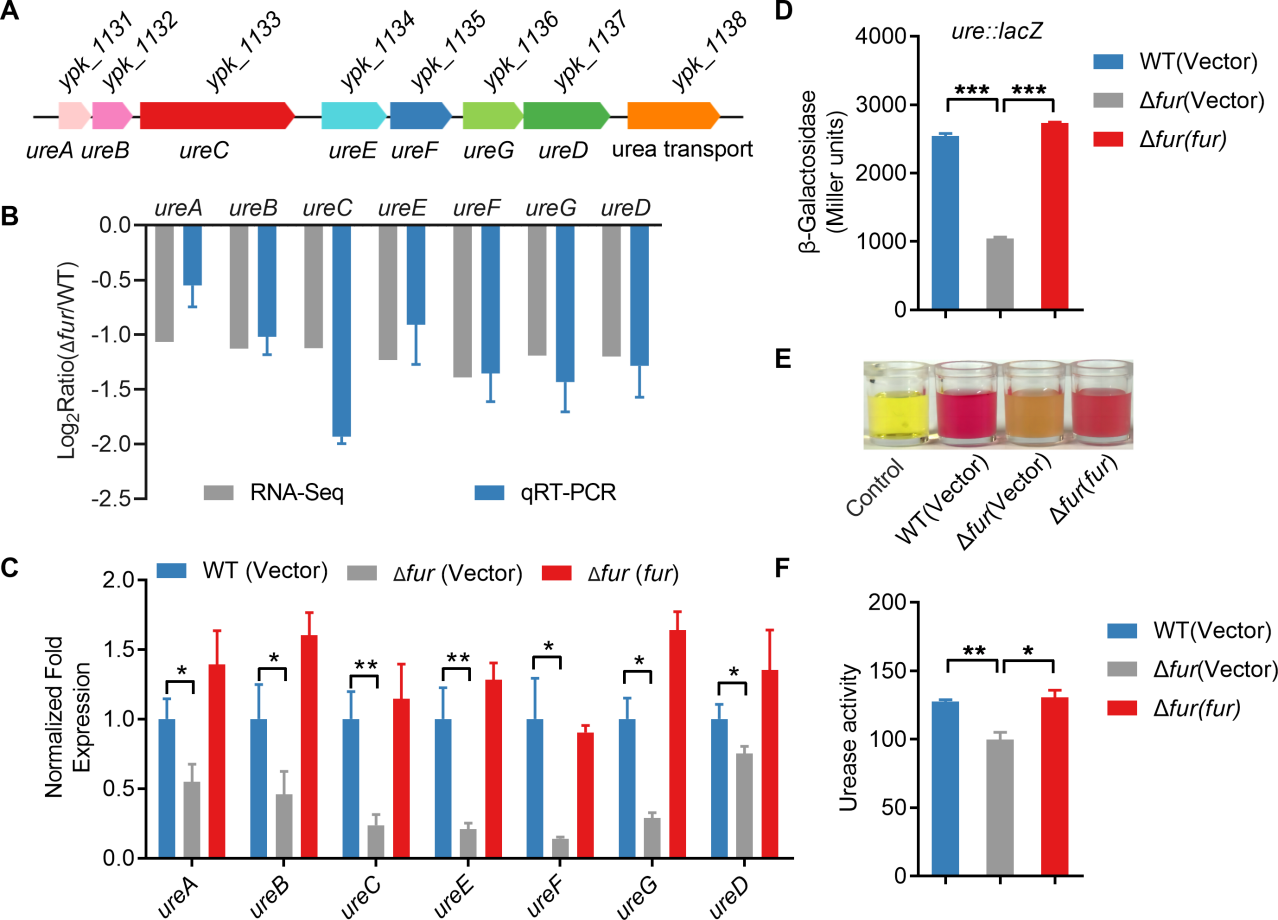
Fur positively regulates urease expression in *Yptb*. (**A**) Gene organization of urease gene cluster in *Yptb*. (**B**) qRT-PCR analysis of urease expression levels. Genes differentially transcribed in *Yptb* Δ*fur* mutant compared with those in the WT were detected by transcriptomic and qRT-PCR analysis. The RNA-seq data were validated by qRT-PCR for seven urease genes (*ureA*, *ureB*, *ureC*, *ureE*, *ureF*, *ureG*, and *ureD*). (**C**) qRT-PCR analysis of mRNA levels of urease in *Yptb* WT, Δ*fur* mutant, and complemented Δ*fur*(*fur*) strains. (**D**) β-galactosidase analysis of urease promoter activity was performed by using the transcriptional P*_ureABC::lacZ_* chromosomal fusion reporter expressed in indicated bacterial strains grown to stationary phase in YLB medium. (**E**) Qualitative analysis of urease activity for indicated bacterial strains. The pH was indicated by phenol red. (**F**) Quantitative analysis of urease activity for WT, Δ*fur,* and Δ*fur*(*fur*) grown in YLB medium. Urease activity is expressed as micromoles of ammonia produced per minute per milligram of protein. Data represent the mean ± SD of three biological replicates, each of which was performed with three technical replicates. *P < 0.05; **P < 0.01; ***P < 0.001.

Urease expression in *Yptb* is positively regulated by the regulator OmpR and negatively regulated by the regulators RovM and CsrA (2, 5). To determine whether Fur regulates urease in *Yptb*, we performed RNA sequencing (RNA-seq)-based transcriptomic analysis and screened them with a threshold of |log_2_Ratio(Δ*fur*/WT)|≥1. Notably, the entire urease gene cluster (Fig. 1A) showed significantly reduced transcription in the Δ*fur* mutant compared to that in the wild type (WT) (Fig. S1). We next validated the transcriptomic data using quantitative real-time polymerase chain reaction (qRT-PCR) analysis of the *ureA*, *ureB*, *ureC*, *ureE*, *ureF*, *ureG*, and *ureD* genes in the urease gene operon. Consistent with the RNA-seq data, the expression of these genes was downregulated in the Δ*fur* mutant (Fig. 1B). Next, we found that the expression level of urease was significantly decreased in the Δ*fur* mutant, and this phenomenon was completely reversed in the complemented strain Δ*fur*(*fur*) (Fig. 1C). We also determined its positive regulation on urease by measuring the transcription activity of chromosomal P*_ureABC_::lacZ* fusions. The *ureABC* promoter activity was significantly decreased in the Δ*fur* mutant, which could be fully restored in the complemented strain Δ*fur*(*fur*) (Fig. 1D). These data above collectively indicate that Fur positively regulates urease expression at the transcriptional level in *Yptb*.

To investigate the influence of Fur on urease production in *Yptb*, we examined the urease activity of the WT, Δ*fur* mutant, and complemented Δ*fur*(*fur*) strains by measuring NH_3_ production using the phenol-hypochlorite method as described previously (29). As shown in Fig. 1E, urease activity was significantly decreased in the Δ*fur* mutant compared to that in the WT, which could be fully restored in the complemented strain Δ*fur*(*fur*). The quantitative urease activity assay further confirmed this result (Fig. 1F). Taken together, these results suggest that Fur positively regulates urease expression in *Yptb*.

### Fur activates urease expression by binding directly to its promoters in *Yptb*

**Fig 2:**
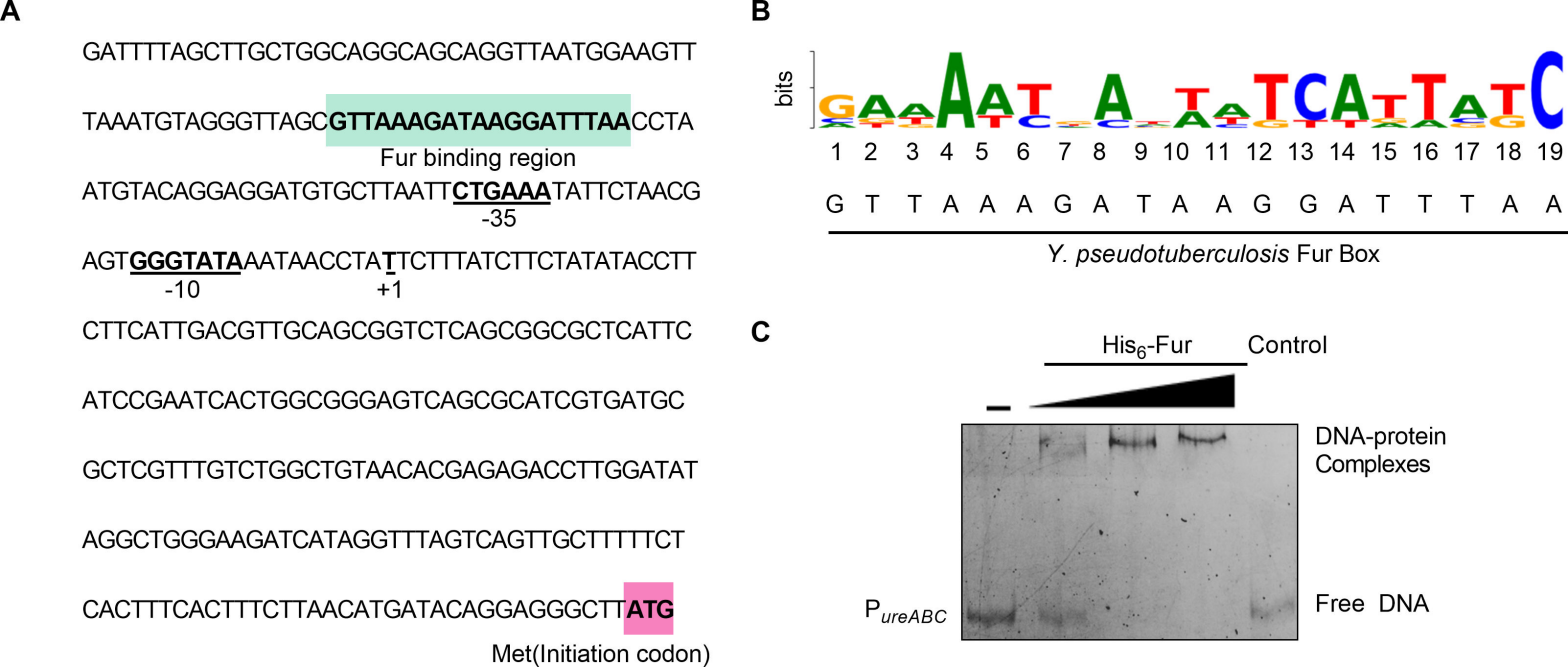
Fur activates urease expression by directly binding to the urease promoter in *Yptb*. (**A**) Identification of the Fur binding site in the promoter region of urease. Putative Fur-binding site identified in green by the online software Virtual Footprint. The ATG start codon of the first ORF of the urease is marked in pink, and the –35 and –10 elements of the urease promoter are underlined. +1 denotes the transcription start point. (**B**) Fur box sequence upstream of *ureABC*. Virtual footprint analysis of the *Yptb* Fur binding sequence. Letters represent the position weight matrix based on the *P. aeruginosa* consensus sequence for Fur binding. The Y-axis represents relative nucleotide probability, and the X-axis represents nucleotide position. *Yptb* Fur box sequence is located at −292 bp of *ureA* and has a probability score of 7.36 (max score = 9.68). (**C**) EMSA was performed to analyze the interaction between His_6_-Fur and the urease promoter (*P_ureABC_*). Increasing amounts of Fur (0.12, 0.34, and 1.2 µM) and 2 ng DNA fragments were used (Control, unrelated BSA protein).

We have shown above that Fur positively regulates urease activity. However, the underlying mechanism is not clear. Given that Fur is a well-known regulator that regulates many iron transport systems by recognizing and binding to conserved Fur boxes within promoter regions (27, 30), we hypothesized that Fur might regulate urease by binding to its promoter. Previous studies have shown that there are three transcriptional units (*ureABC*, *ureEF,* and *ureGD*) in the urease cluster (7), with a promoter region located upstream of each unit in *Yptb* (5). We further analyzed the promoters of the urease *ureABC* operon and revealed a putative Fur-binding site at the promoter (Fig. 2A). Further analysis showed that the putative Fur-binding site is highly similar to the Fur box in *P. aeruginosa* (Fig. 2B). To further investigate the direct regulation of urease expression by Fur, we examined the interaction between Fur and the promoter of *ureABC* using electrophoretic mobility shift assay (EMSA). First, a DNA fragment of 350 bp upstream of *ureA*, designated P*_ureABC_* was amplified and used as the probe for Fur binding. As shown in Fig. 2C, incubation of the P*_ureABC_* probe with His_6_-Fur resulted in the formation of DNA-protein complexes. Meanwhile, the specific interaction was also confirmed, as an excess of the unrelated protein bovine serum albumin (BSA) failed to form the protein-DNA complexes (Fig. 2C). Thus, we have shown that Fur activates urease expression by binding directly to its promoters in *Yptb*.

### Fur-mediated expression of urease responds to Mn^2+^ under low nutrient conditions

**Fig 3:**
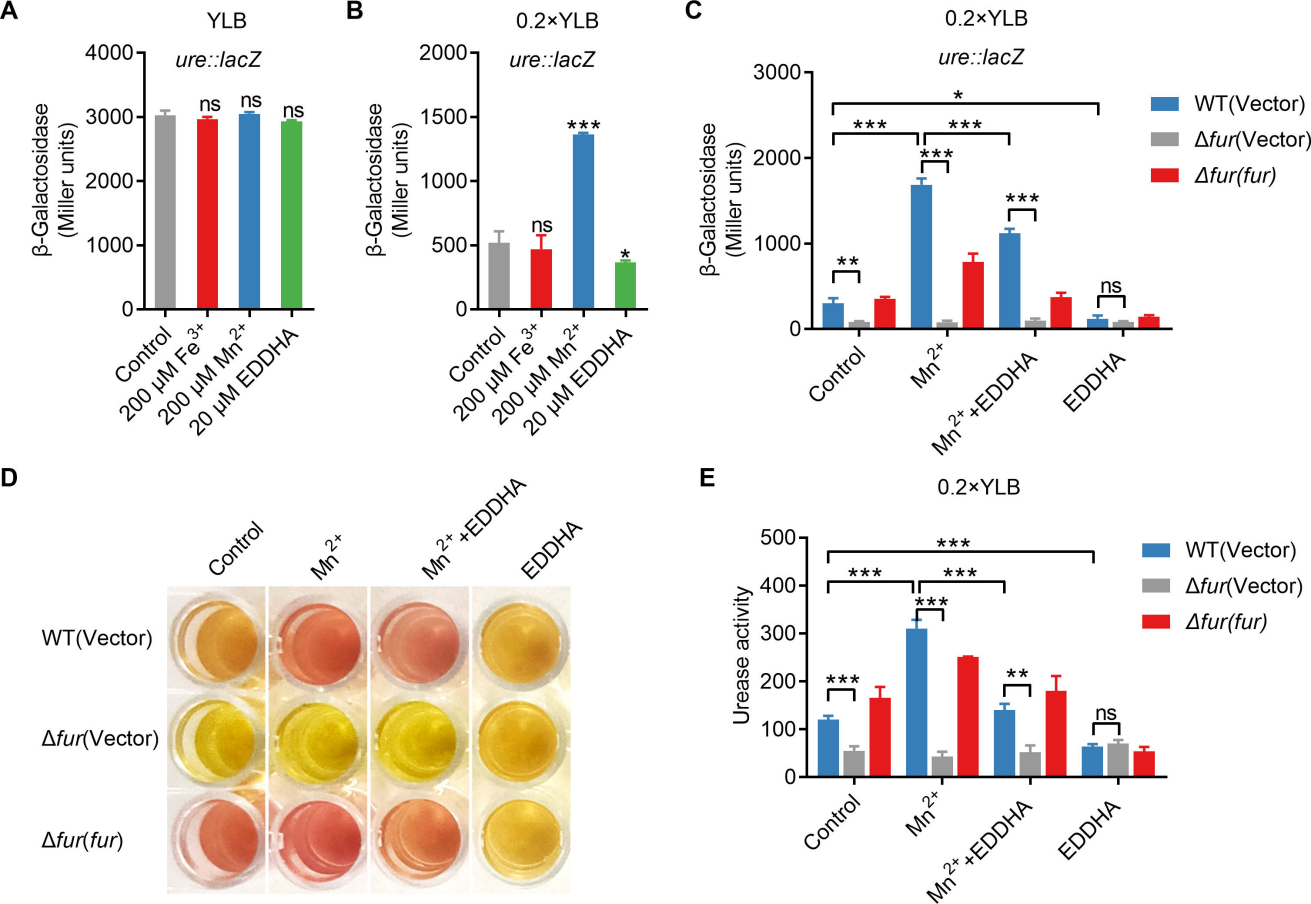
Fur-mediated expression of urease responds to Mn^2+^ under low nutrient conditions. (**A**) Urease expression is not influenced by Fe^3+^ or Mn^2+^ under relatively rich-nutrient YLB medium. β-galactosidase analysis of urease (*ureABC*) promoter activities was performed in *Yptb* WT strains grown to stationary phase in YLB medium with or without 200 µM Fe^3+^, 200 µM Mn^2+^, and 20 µM EDDHA. (**B**) Urease expression is induced by Mn^2+^ under low nutrient conditions. β-galactosidase analysis of urease (*ureABC*) promoter activities was performed in *Yptb* strains grown to stationary phase in 0.2 × YLB medium with or without 200 µM Fe^3+^, 200 µM Mn^2+^, and 20 µM EDDHA. (**C-E**) Fur-mediated expression of urease is responsive to Mn^2+^ under low nutrient conditions. The related *Yptb* WT, Δ*fur*, and Δ*fur*(*fur*) strains, either harboring P*_ureABC::lacZ_* (**C**) or not (**D and E**), were grown to stationary phase in 0.2 × YLB medium supplemented with 200 µM Mn^2+^, 200 µM Mn^2+^ + 20 µM EDDHA (Mn^2+^ + EDDHA), or 20 µM EDDHA. The expression of the reporter was measured (**C**).The urease activity of the indicated bacterial strains was analyzed qualitatively (**D**) and quantitatively (**E**). The pH was indicated using phenol red. Urease activity is expressed as micromoles of ammonia produced per minute per milligram of protein. The difference in urease expression in WT, Δ*fur*, and Δ*fur*(*fur*) strains grown to stationary phase in 0.2 × YLB medium with or without 200 µM Mn^2+^ or 20 µM EDDHA by β-galactosidase analysis of urease promoter activities, qualitative and quantitative urease activity assay. Data represent the mean ± SD of three biological replicates, each of which was performed with three technical replicates. *P < 0.05; **P < 0.01; ***P < 0.001; ns, not significant.

As a key protein involved in bacterial iron homeostasis, the Fur protein plays a vital role in various metabolic regulations (31, 32). Previous studies have shown that Fur-mediated T6SS regulation is linked to Mn^2+^ levels in *Yptb* (21). Therefore, we first examined the effect of varying Fe^3+^ and Mn^2+^ concentrations on urease expression. As shown in Fig. 3A, urease expression in WT was not affected by Fe^3+^ or Mn^2+^ concentration in a relatively nutrient-rich YLB medium. Remarkably, when the nutrient content of the medium was reduced by a factor of 5 (0.2 × YLB), the transcriptional activity of urease increased significantly in Mn^2+^-replete conditions rather than in Fe^3+^-replete conditions. Furthermore, the addition of ethylenediamine-*N, N’*-bis(2-hydroxyphenylacetic acid) (EDDHA), a Mn^2+^ chelator, inhibited this increase, indicating that urease expression was unresponsive to Fe^3+^ but showed sensitivity to Mn^2+^ (Fig. 3B). To determine whether the positive regulation of urease by Fur is sensitive to Mn^2+^ concentration in *Yptb*, we determined the expression of urease in WT, Δ*fur*, and Δ*fur*(*fur*) using chromosomal P*_ureABC::lacZ_* fusion reporter analysis at different Mn^2+^ concentrations. The results indicate that the presence of Mn^2+^ activates Fur-regulated urease. In the absence of Mn^2+^, there was no significant difference in urease transcription activity between WT and Δ*fur* mutant under 0.2 × YLB conditions (Fig. 3C). Further analysis by quantitative and qualitative assessment of urease activity supported the results obtained from the β-galactosidase assay (Fig. 3D and E). Collectively, these results suggest that Fur-mediated expression of urease responds to Mn^2+^, rather than Fe^3+^, under low nutrient conditions.

### Urease increases the ability of *Yptb* to resist environmental stress and biofilm formation

**Fig 4:**
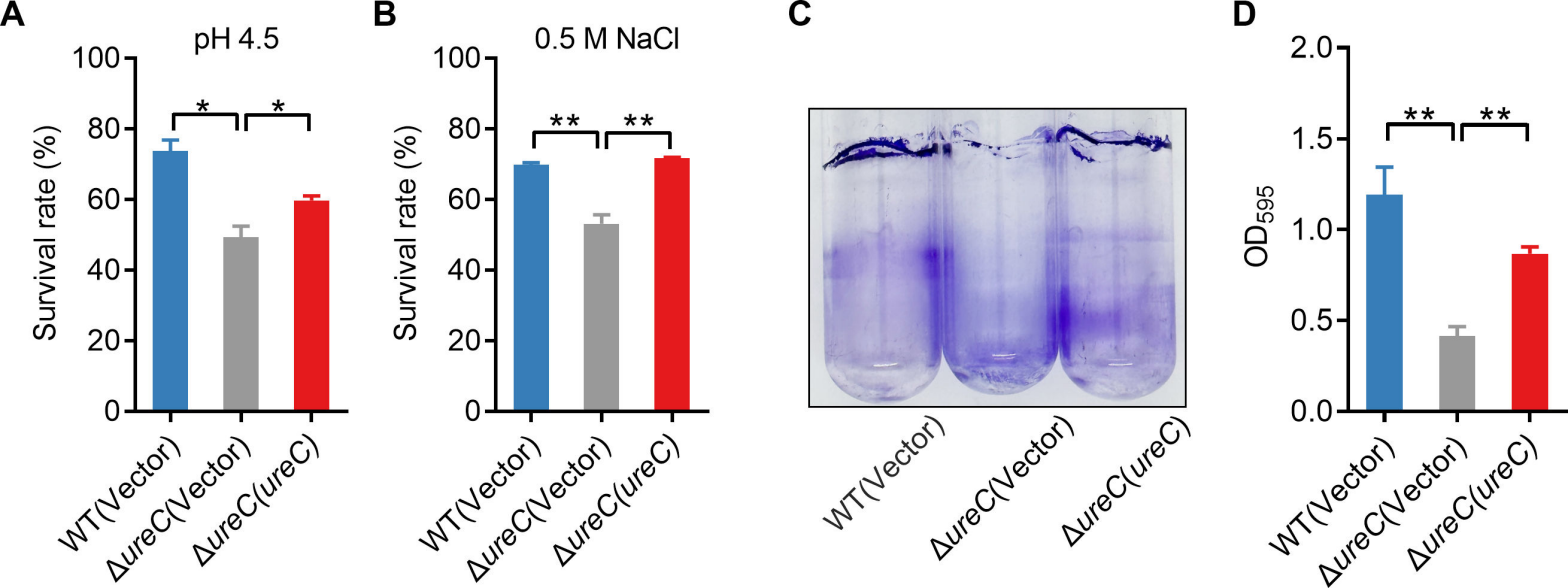
Urease increases the ability of *Yptb* to resist environmental stress and biofilm formation. (**A and B**) UreC is involved in combating acid and osmotic stress. Survival rates of *Yptb* WT, Δ*ureC* mutant, and complemented Δ*ureC*(*ureC*) strains after exposure to pH 4.0 (**A**) or 0.5 M NaCl (**B**) for 40 min. (**C**) UreC influences the biofilm formation of *Yptb*. The saturated bacterial cultures were diluted 100-fold in a fresh YLB medium. After 3 days of shaking at 150 rpm at 26°C, biofilm formation of the strains was determined by crystal violet staining and quantified using optical density measurement. Data represent the mean ± SD of three biological replicates, each of which was performed with three technical replicates. *P < 0.05; **P < 0.01.

Urease increases the resistance of many pathogenic bacteria, such as *H. pylori* and *B. abortus*, to an acidic pressure environment (33, 34). To further explore whether urease plays a crucial role in enhancing the survival of *Yptb* under stress conditions, we created the *ureC* (key urease structural gene) deletion mutant and thus determined the viability of the urease mutant after challenge with pH 4.5 EG buffer and 0.5 M NaCl for 40 min. The results showed that the survival rates of the Δ*ureC* mutant were significantly more sensitive to acid and osmotic stress than the WT (Fig. 4A and B). Meanwhile, the survival rates of the complemented Δ*ureC*(*ureC*) strain were almost completely restored to the WT levels, supporting a role for urease in combating acid and osmotic stress. Collectively, these data demonstrate that urease contributes to the environmental stress in *Yptb*.

Biofilm is an important strategy adopted by pathogenic bacteria to survive in harsh environments and during host infection (35). *Yptb* is capable of forming biofilm, which contributes to environmental survival and virulence (36). We examined the biofilm-forming capacity of WT and urease biosynthetic mutants using the crystal violet assay. As shown in Fig. 4C and D, the Δ*ureC* mutant showed an obvious defect in biofilm formation compared to WT, and the biofilm-formation capacity was restored to WT levels by complementation with *ureC*. Thus, these results indicate that urease plays an important role in resisting adverse stresses and biofilm formation in *Yptb*.

### Urease slightly enhances the survival advantage of *Yptb* in infected mice

**Fig 5:**
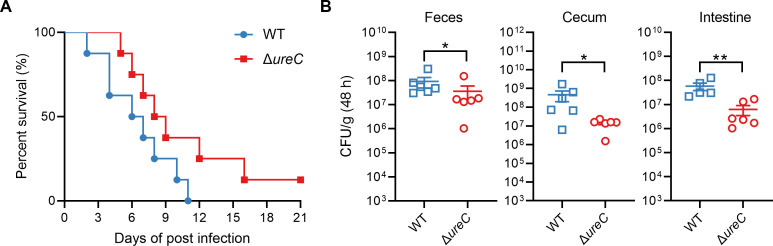
Urease slightly enhances the survival advantage of *Yptb* in infected mice. (**A**) and (**B**) *Yptb* WT and Δ*ureC* strains grown in YLB were washed twice in sterilized PBS and used for orogastric infection of 6-week-old female BALB/c mice using a ball-tipped feeding needle. For survival assays, 1 × 10^9^ bacteria of each strain were applied to different groups of mice (*n* = 9/strain), and the survival rate of the mice was determined by monitoring the survival daily for 21 days (**A**). Enumeration of *Yptb* WT and Δ*ureC* strain burdens in the feces, cecum, and intestine of infected BALB/c mice at 48 h post-infection by CFU assays (*n* = 6) (**B**). Similar results were obtained in three independent experiments, and the data shown are from one representative experiment done in triplicate (**A**) and (**B**). The statistical significances were determined by the Mann–Whitney test (**B**). Data represent the mean ± SD of three biological replicates, each of which was performed with three technical replicates. The statistical significance was determined by the Mann-Whitney test. *P < 0.05; **P < 0.01.

The urease activity and motility of the pathogen are relevant pathogenic factors for the initial bacterial colonization and survival in the host (37), which led us to further investigate whether urease was involved in the virulence. BALB/c mice were orogastrically infected with the WT and Δ*ureC* mutant, respectively, and the survival rate of each group was analyzed. The results showed that infection with the WT resulted in 100% death within 3 weeks of infection, and the lethality rates slightly but substantially decreased in the Δ*ureC* mutant infected group (Fig. 5A). Bacterial loads recovered from the feces, cecum, and intestine at 48 h post-infection with *Yptb* strains were then counted. Consistently, mice infected with the Δ*ureC* mutant had significantly lower loads compared to WT-infected mice (Fig. 5B). Taken together, these results suggest that urease contributes to the virulence and survival of *Yptb* in infected mice.

## DISCUSSION

Urease is crucial for bacterial colonization in the gastrointestinal tract as it breaks down urea into CO_2_ and NH_3_, which helps to neutralize highly acidic environments (6). Studies have shown that urease functions similarly in the acid resistance system of *Yptb* and is regulated by the global factor RovM (2). Recent reports have found that urease activity is regulated by Fur in *E. coli* and *H. pylori* (25, 26). However, it remains unclear whether Fur regulates urease expression in *Yptb*. In this study, we show that urease from *Yptb* is positively regulated by Fur in an Mn^2+^-dependent manner, which functions to combat acid and osmotic stress and enhance biofilm formation and plays a crucial role in virulence and survival in infected mice.

Bacteria can sense changing external cues, including pH and iron, to control the expression of urease through a number of transcriptional regulators that control their environmental adaptability and virulence (9, 11, 27). For example, *H. pylori* senses the acid signal to induce urease expression through the ArsRS two-component system and the NikR regulatory protein (38). Urease expression in *S. aureus* is regulated by CcpA, Agr, and CodY in response to changes in metabolic flux (9). Our previous studies have shown that *Yptb* senses acid and nutrient conditions to regulate urease expression through OmpR, CsrA, and RovM (2, 5), and here we further investigate the role of Fur in regulating urease expression. Our data show that Fur positively regulates urease expression and activity in *Yptb* (Fig. 1). Although Fur-mediated urease regulation has been studied in other pathogenic bacteria, the regulation mechanisms differ significantly from our findings. For example, Fur positively regulates urease activity in *K. pneumoniae*, probably by controlling intracellular iron and nitrogen concentrations, but not at the transcriptional level (28). In *H. pylori*, mutation of *fur* does not affect urease expression at the transcriptional level or the basal protein level in the medium without nickel. However, nickel, but not iron, manganese, or zinc, induces urease enzyme activity, and the nickel-responsive induction of urease was clearly diminished in *H. pylori* (24). *H. hepaticus* sense iron signal to induce urease expression through Fur (27). Our investigations demonstrate that Fur not only activates the expression of the entire urease gene cluster at the transcriptional level by binding directly to the urease promoter but also positively regulates urease activity in *Yptb* (Fig. 1 and 2). Interestingly, Mn^2+^, but not Fe^3+^, can induce urease expression under low nutrient conditions. Although Fur is an iron-responsive regulator (39), our previous study showed that Fur of *Yptb* is a dual regulator that senses Mn and Fe (21). Further investigations indicate that Fur-mediated expression of *Yptb* urease is responsive to Mn^2+^ under low nutrient conditions, revealing a novel regulatory pathway of the bacterial urease system. While urease contributes to the survival and virulence of *Yptb* in infected mice (Fig. 5), we speculate that *Yptb* may regulate urease levels via Fur after sensing Mn^2+^ concentration upon infection of the host cell, thereby influencing bacterial physiology and pathogenicity.

Urease activity in *Yptb* is subject to complex regulation by different transcriptional regulators, including OmpR, CsrA, RovM, and Fur (2, 5), raising the possibility that urease activity is coordinated by multiple transcriptional regulatory pathways. Each of these regulators responds to different environmental signals and stresses, enabling *Yptb* to fine-tune urease expression in response to changing conditions. For instance, the two-component regulator OmpR positively regulates urease activity in a pH-dependent manner to enhance bacterial acid survival in *Yptb* (5). Under nutrient-rich conditions, RovM and CsrA are suppressed, while the urease activator OmpR is activated, leading to high urease expression. Conversely, under nutrient-limited conditions, OmpR is deactivated, while CsrA is upregulated and RovM is activated, resulting in the repression of urease expression in *Yptb* (2). Furthermore, our data indicate that Fur-activated urease expression is responsive to Mn^2+^ under low nutrient conditions (Fig. 1 and 3). The interconnectivity of these pathways suggests that OmpR, CsrA, RovM, and Fur may form a complex regulatory network to coordinately control urease expression, which is further involved in a variety of important physiological functions. While the specific mechanisms and interconnections of this complex regulatory network in regulating urease in *Yptb* remain unclear, we believe that this intricate regulation reflects the adaptive strategies employed by *Yptb* to optimize urease production and enhance its survival and pathogenicity in response to changing and adverse conditions.

Bacteria have been exposed to a variety of stressful conditions throughout their evolution, including acidity, high osmotic pressure, temperature extremes, and fluctuating nutrient availability. Pathogenic bacteria, in particular, have developed defense strategies including proton pumps, urease, arginine deiminase, sigma factors, and biofilm formation to thrive in acidic environments where they frequently infect hosts (37, 40–43). For example, *S. aureus* utilizes urease to maintain pH balance, protecting itself from reactive oxygen species (ROS) and preserving cell integrity (9). *H. pylori*, a prevalent human pathogen responsible for gastric diseases and potentially gastric cancer, employs ureases to convert urea into CO_2_ and NH_3_, adjusting its urease permeability in response to environmental pH changes (44). By contrast, *K. pneumoniae*, which lacks the urease core gene *ureC*, struggles to survive in highly acidic environments at pH 2.5, highlighting the critical role of urease in acid survival (28). Our findings demonstrate that urease enhances *Yptb*’s ability to withstand acid and osmotic stress (Fig. 4), suggesting that urease is a vital mechanism for *Yptb*’s survival under stressful conditions.

The urease system is not only a strategy for obtaining nutrients but also an important tool used by pathogenic bacteria against the host (37, 45). Urease enables *H. pylori* to obtain nutrients and colonize within the host (46, 47). *Cryptococcus neoformans* utilizes urease to cause brain infections in humans (48), where the absence of urease not only inhibits growth but also reduces survival (49). In addition, *Aspergillus fumigatus* spores lacking urease are more susceptible to macrophage-mediated killing (50). Recent research has demonstrated that UreC can interfere with host DNA repair in *Mycobacterium tuberculosis* (51). Therefore, our investigation aimed to assess the effect of urease of the *Yptb* on virulence. Our results showed that mice infected with the Δ*ureC* mutant had a lower mortality rate and bacterial load compared to WT-infected mice. However, there is a different report on whether urease is involved in the virulence of *Yptb* in a previous study (52), probably due to differences in strain characteristics and experimental conditions in these studies. The pYV plasmid, which encodes a key virulence factor, is present in some but not all *Yptb* strains (53). Riot et al. performed mouse infection experiments using *Yptb* IP2777 (pYV+) WT and Δ*ureB*, but we used *Yptb* YPIII (pYV-) WT and Δ*ureC* to infect mice. Their results have shown that there is no significant difference in Peyer’s patches, mesenteric lymph nodes, spleen, and liver between the CFU counts of mice infected with 1 × 10^8^ CFU *Yptb* IP2777 (pYV+) WT or the Δ*ureB* mutant strains. Our data indicate that mice infected with 1 × 10^9^ CFU *Yptb* YPIII (pYV-) Δ*ureC* mutant had significantly lower loads in the feces, cecum, and intestine compared to WT-infected mice (Fig. 5B), suggesting that urease slightly enhances the survival advantage of *Yptb* YPIII in infected mice. These observations prompt the hypothesis that urease, as a minor virulence factor, may contribute to the virulence and survival of *Yptb* in infected mice in the absence of the key virulence factor encoded by pYV. Moreover, we believe that it is an insufficient argument to conclude that urease is not involved in bacterial virulence only based on the observation that there is no difference in the colonization ability between *Yptb* IP2777 (pYV+) WT and Δ*ureB*. For example, previous research has demonstrated that the CNF_Y_ toxin does not exert its virulence by enhancing bacterial colonization in mice, but rather by altering the composition of the gut microflora and inducing host inflammatory responses (54). Here, we further analyzed the survival rate of mice after infection with 1 × 10^9^ CFU *Yptb* YPIII (pYV-) WT and Δ*ureC* mutant (Fig. 5A), confirming that urease enhances bacterial virulence in a murine model. Thus, the urease produced by *Yptb* YPIII appears to act as a virulence factor. Overall, our results reveal that urease from *Yptb* is directly regulated by Mn^2+^ via Fur, which combats acid and osmotic stress and enhances bacterial virulence and fitness.

## MATERIALS AND METHODS

### Bacterial strains, plasmid constructions, and growth conditions

The bacterial strains and plasmids used in this study are listed in Table S1. The primers used are detailed in Table S2. *E. coli* strains were grown in Luria-Bertani (LB) broth (1% tryptone, 0.5% yeast extract, and 1% NaCl) with appropriate antibiotics at 37°C, while *Yptb* strains were cultured in Yersinia-Luria-Bertani (YLB) broth (1% tryptone, 0.5% yeast extract, and 0.5% NaCl) or M9 minimal medium (Na_2_HPO_4_, 6 g L^−1^; KH_2_PO_4_, 3 g L^−1^; NaCl, 0.5 g L^−1^; NH_4_Cl, 1 g L^−1^; MgSO_4_, 2 mM; CaCl_2_, 0.1 mM; glucose 0.2%, pH 7.0) at 26°C with appropriate antibiotics when necessary. All chemicals were of Analytical Reagent Grade purity or higher. The *Yptb* YPIII strain was the parent of all derivatives used in this study. To complement the Δ*ureC* mutant, we used primers *ureC*-F-BglII/*ureC*-R-SalI to amplify the *ureC* gene from the *Yptb* genomic DNA. The PCR product of *ureC* was then digested with BglIII/SalI and inserted into the BamHI/SalI sites of pKT100, resulting in pKT100-*ureC*. Furthermore, the complementary plasmid was introduced into the Δ*ureC* mutant by electroporation. The integrity of the insert in all constructs was confirmed by DNA sequencing. Cell growth was monitored by measuring the optical density (OD) at 600 nm. Antibiotics were added at the following concentrations: nalidixic acid, 20 µg mL^−1^; kanamycin, 50 µg mL^−1^; chloramphenicol, 20 µg mL^−1^.

### RNA-seq assay

RNA-seq experiment Total RNA was extracted from *Yptb* WT and the Δ*fur* mutant grown in the YLB at 26°C with shaking (220 rpm) to a final OD_600_ of approximately 1.6, using bacteria total RNA isolation kit (TIANGEN, Beijing, China). RNA extraction, library construction, and RNA-seq were commissioned by BGI Genomics (Shenzhen, China). Differential expression analysis was performed using the NOIseq method (Sonia Tarazona 2100). *P*-values were adjusted using the Benjamini and Hochberg method. A corrected *P*-value of 0.05 and log_2_(fold change) of 0.8 were set as the threshold for significantly differential expression. Gene Ontology (GO) enrichment analysis of differentially expressed genes was implemented by the GOseq R package, in which gene length bias was corrected. GO terms with corrected *P*-values less than 0.05 were considered significantly enriched by differentially expressed genes. The data have been deposited under bioProject accession number PRJNA632467.

### qRT-PCR

Bacteria cells were harvested during the mid-exponential phase, and RNA was extracted using the RNA prep Pure Cell/Bacteria Kit and treated with RNase-free DNase (TIANGEN, Beijing, China). First-strand cDNA was reverse transcribed from 1 µg of total RNA with the TransScript First-Strand cDNA Synthesis SuperMix (TransGen Biotech, Beijing, China). qRT-PCR was performed in the CFX96 Real-Time PCR Detection System (Bio-Rad, USA) with TransStart Green qPCR SuperMix (TransGen Biotech, Beijing, China). For all primer sets (Table S2), the following cycling parameters were used: 95°C for 30 s followed by 40 cycles of 94°C for 15 s, and 50°C for 30 s. For standardization of results, the relative abundance of 16S rRNA was used as the internal standard. All samples were analyzed in triplicate, and the expression of target genes was calculated as relative fold values using the 2^−ΔΔCT^ method. These assays were performed in triplicate at least three times, and error bars represent the standard error of the mean.

### β-galactosidase assays

The *lacZ* fusion reporter vector pDM4-P*_ureABC_::lacZ* was transformed into *E. coli* S17-1*λ pir* and mated with *Yptb* strains as described previously (55). The *lacZ* fusion reporter strains were grown to stationary phase in YLB or 0.2 × YLB at pH 7.0 under 26°C, and β-galactosidase activity was assayed using ONPG (o-Nitrophenyl β-D-galactopyranoside) as the substrate. These assays were performed in triplicate at least three times, and error bars represent standard deviations.

### Urease qualitative assay

Phenol red was used as a pH indicator to observe pH changes caused by urease hydrolysis. The qualitative urease tests were carried out according to the methods (29). In summary, the presence of NH_3_ results in a pH shift from neutral to alkaline in the medium, using phenol red as the pH indicator. This change is visually indicated by a change in the color of the medium from yellow to pink. After centrifugation at 5,000 g for 10 min, the cells are resuspended in 2 mL of urease test solution (0.5% [wt/vol] NaCl, 0.2% [wt/vol] KH_2_PO_4_, 0.2% [wt/vol] urea, 0.002% [wt/vol] phenol red), and incubated for 4 h to observe the color of the medium and to determine urease activity.

### Urease quantitative assay

Urease activity was quantified by determining the rate of NH_3_ production from the hydrolysis of urea (2). Briefly, bacteria were cultured at 26°C and harvested at the late exponential phase. Bacterial cells were washed twice with PBS buffer and then resuspended. Add 5 µL of bacterial suspension to 40 µL of test buffer (0.1% [wt/vol] cetyl dimethyl ammonium bromide [CTAB], 0.6% [wt/vol] NaCl, 100 mM citrate, 5 mM urea, pH 6.0) and incubate with stirring. Add 100 µL of phenol nitroprusside followed by 100 µL alkaline hypochlorite to terminate the reaction. After standing for 30 min at room temperature, the absorbance at 635 nm was measured, and the protein concentration was quantified by the Bradford method using BSA as the standard scale. NH_3_ concentration was determined by constructing a standard curve using freshly prepared defined concentrations of NH_4_Cl in the same buffer. Urease activity is expressed as micromoles of NH_3_ produced per minute per milligram of protein.

### Overexpression and purification of recombinant protein

The overexpression and purification of recombinant proteins were conducted according to established methods (56). To express and purify soluble His_6_-tagged recombinant proteins, the plasmid pET28a-*fur* was transformed into BL21(DE3). Bacteria were cultured at 37°C in LB medium to an OD_600_ of 0.5, shifted to 24°C, induced with 0.2 mM IPTG, and then cultivated for an additional 12 h at 24°C. Harvested cells were disrupted by sonication, and proteins were purified with the His•Bind Ni-NTA resin (Novagen, Madison, WI) according to the manufacturer’s instructions. Eluted recombinant proteins were dialyzed against buffer (50 mM Tris, 137 mM NaCl, 10% glycerol, pH 7.5) at 4°C. The resulting proteins were stored at −80°C until use. Protein concentrations were determined using the Bradford assay according to the manufacturer’s instructions (Bio-Rad, Hercules, CA) with BSA as standard.

### Electrophoretic mobility shift assay

The EMSA was performed according to Zuo and colleagues (21). The P*_ureABC_* fragment was amplified from the *Yptb* YPIII genome using the primers P*_ureABC_*-F and P*_ureABC_*-R. Increasing concentrations of purified His_6_-Fur (0.12, 0.34, and 1.2 µM) were incubated with 2 ng DNA probes in EMSA buffer (20 mM Tris, pH 7.4, 4 mM MnCl_2_, 100 mM NaCl, 1 mM dithiothreitol, and 10% glycerol). After incubation for 30 min at room temperature, the binding reaction mixture was subjected to electrophoresis on a 6% native polyacrylamide gel containing 5% glycerol in 0.5 × TBE (Tris-borate-EDTA) electrophoresis buffer, and the DNA probe was detected using SYBR Green. As a negative control, an irrelevant protein (BSA) was included in the binding assay.

### Bacterial survival assays

Mid-exponential phase *Yptb* strains grown in YLB medium were collected, washed, and diluted 50-fold into M9 medium and treated with NaCl (0.5 M) or pH 4.5, respectively, at 26°C for 40 min. After treatment, the cultures were serially diluted and plated onto YLB agar plates, and colonies were counted after 36 h growth at 26°C. Percentage survival was calculated by dividing the number of colony-forming units (CFU) of stressed cells by the number of CFUs of cells without stress. All these assays were performed in triplicate at least three times.

### Biofilm formation assay

Biofilm formation was assessed as previously described (57). *Yptb* strains were cultivated in YLB broth and subsequently transferred to 3 mL of M9 medium, shaken at 150 rpm at 26°C. Overnight bacterial cultures were diluted 100-fold in 5 mL of fresh YLB medium, with appropriate antibiotics added when necessary. After vertical incubation for 3 days at 150 rpm and 26°C, the bacterial cultures were removed following OD_600_ measurements, and the test tubes were washed twice with fresh M9. Cells adhering to the test tubes were stained with 0.1% crystal violet for 30 min and then washed twice with M9. The cell-bound dye was eluted in 6 mL of 95% ethanol, and the absorbance of the eluted solution was measured at 590 nm using a microplate reader.

### Murine infection assay

The protocol for this study was approved by the Animal Welfare and Research Ethics Committee of Northwest A&F University (Protocol number: XN2023-1004). Six-week-old female BALB/c mice were obtained from SPF Biotechnology Co., Ltd (Beijing, China) and housed in a controlled environment with a temperature of 24 ± 2°C, the humidity of 50 ± 10%, an air flow rate of 35 exchanges per hour, and a light-dark cycle of 12 h each. Mid-exponential phase *Yptb* strains were grown in YLB medium at 26°C, washed twice in sterilized PBS, and used for intragastric or intraperitoneal infection of 6- to 8-week-old female BALB/c mice. For survival assays, 1 × 10^9^ bacteria from each strain were orally gavaged to different groups of mice, and the survival rate was monitored daily for 21 days. To analyze the bacterial load in the feces, fecal samples were collected from individual living mice at 48 h post-infection, weighed, and homogenized in PBS. For the assessment of bacterial load in the cecum and intestine, mice were sacrificed via carbon dioxide asphyxiation followed by cervical dislocation at 48 h post-infection. The tissues were then weighed and homogenized in PBS, and serial dilutions of the homogenates were plated on YLB plates containing 20 µg mL^−1^ nalidixic acid. The CFUs were counted and reported as CFU per gram of organ or tissue.

### Bioinformatics analyses

Sequence alignment and database searches were conducted using the BLAST server on the National Center for Biotechnology Information (NCBI) website (https://www.ncbi.nlm.nih.gov/).

### Statistical analysis

Experimental data analyzed for significance were analyzed using GraphPad Prism 8 (GraphPad Software, San Diego California USA). The *P* values for mouse survival were calculated using the Log-rank (Mantel-Cox) test. The *P* values for bacterial CFU in mouse tissues were calculated using the Mann-Whitney test (I). Statistical analyses for the rest of the assays were performed using unpaired two-tailed Student’s *t*-test. Error bars represent ±SD. *P < 0.05; **P < 0.01; ***P < 0.001.

## Data Availability

All data sets generated for this study are included in the article/Supplementary Information.

## SUPPLEMENTAL MATERIAL

Supplemental material spectrum.02756-24-s0001.docx Fig. S1; Tables S1 and S2.

ASM does not own the copyrights to Supplemental Material that may be linked to, or accessed through, an article. The authors have granted ASM a non-exclusive, world-wide license to publish the Supplemental Material files. Please contact the corresponding author directly for reuse.
