# Supplementary material for: Fur-regulated urease contributes to the environmental adaptation of Yersinia pseudotuberculosis
Source: Microbiol Spectr. 2025 Feb 25;13(4):e02756-24. doi: 10.1128/spectrum.02756-24 (PMC11960103; doi:10.1128/spectrum.02756-24)
Supplement: Supplemental material — Fig. S1; Tables S1 and S2. [file spectrum.02756-24-s0001.docx]

**Supplementary Information**

**Fur-regulated urease contributes to environmental adaptation of *Yersinia pseudotuberculosis***

**This PDF file includes:**

**Supplementary Figure 1**

**Supplementary Tables 1-2**

**Supplementary References**


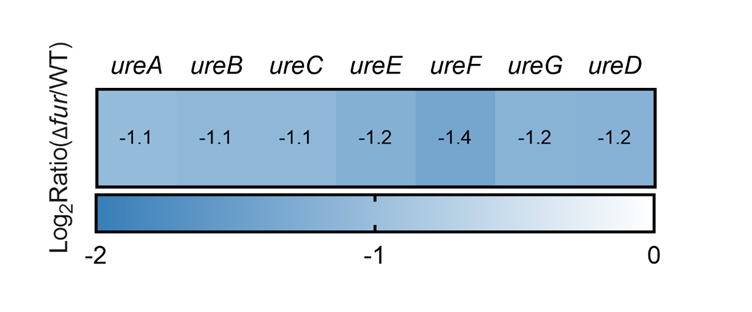


# Fig. S1 Heat map of transcriptomic analysis.

Heat map of transcriptomic analysis. All genes in the urease cluster differentially transcribed in the *Yptb* Δ*fur* mutant compared with those in the WT were detected by RNA-seq data analysis.

**Table 1 Bacterial strains and plasmids.**

| **Strain or plasmid** | **Relevant characteristics** | **Reference** |
| --- | --- | --- |
| ***E. coli*** | | |
| BL21(DE3) | Host for expression vector pET28a or pGEX-6p-1 | Novagen |
| S17-1*λ pir* | *λ-pir* lysogen of S17-1, *thi pro hsdR hsdM^+^ recA* RP4 -Tc::Mu-Km::Tn7 | ([1](#_ENREF_1)) |
| DH5α | FΦ80Δ*lacZ*ΔM15/Δ(*lacZYA-argF*)*U169recA1 endA1 hsdR17* | Novagen |
| ***Y. pseudotuberculosis*** | | |
| WT | Wild-type *Yersinia pseudotuberculosis* YPIII (*Yptb*), Nal^r^ | ([2](#_ENREF_2)) |
| Δ*fur* | *fur* gene deleted in *Yptb*, Nal^r^ | ([3](#_ENREF_3)) |
| Δ*ureC* | *ureC* gene deleted in *Yptb*, Nal^r^ | ([4](#_ENREF_4)) |
| WT(Vector) | WT(Vector) containing pKT100, Nal^r^, Km^r^ | ([3](#_ENREF_3)) |
| Δ*fur*(Vector) | Δ*fur* containing pKT100, Nal^r^, Km^r^ | ([3](#_ENREF_3)) |
| Δ*fur*(*fur*) | Δ*fur* containing pKT100-*fur*, Nal^r^, Km^r^ | ([3](#_ENREF_3)) |
| Δ*ureC*(Vector) | Δ*ureC* containing pKT100, Nal^r^, Km^r^ | This study |
| Δ*ureC*(*ureC*) | Δ*ureC* containing pKT100-*ureC*, Nal^r^, Km^r^ | This study |
| **Plasmid** |  |  |
| pKT100 | Cloning vector, p15A replicon, Km^r^ | ([5](#_ENREF_5)) |
| pKT100-*ureC* | *ureC* expressed in pKT100, Km^r^ | This study |
| pDM4-P*_ureABC_*::*lacZ* | For *ureABC* promoter fusion to *Yptb* chromosome, Cm^r^ | ([6](#_ENREF_6)) |
| pET28a | Expression vector with N-terminal hexahistidine affinity tag, Km^r^ | Novagen |
| pET28a-*fur* | pET28a carrying fur coding region of, Km^r^ | ([3](#_ENREF_3)) |

*Nal^r^, Km^r^, and Cm^r^ represent resistance to Naladixic acid, Kanamycin, and Chloramphenicol at 20, 50, and 20 μg mL^-1^, respectively.

**Table 2 Primers used in this study.**

| **Primers** | **5’-3’ sequence** | **Function** |
| --- | --- | --- |
| *ureC-*F*-*BglII | GAAGATCTATGCCTCAAATTTCTCGG | To generate pKT100-*ureC* |
| *ureC-*R*-*SalI | ACGCGTCGACGCCAAAGAAATAGCGCTG |  |
| P*_ureABC_*-F | GATTTTAGCTTGCTGGCAGG | EMSA |
| P*_ureABC_*-R | CTGCATAAGCCCTCCTGTATCA |  |
| Q16S-F | CTAGCGATTCCGACTTCAT | qRT-PCR |
| Q16S-R | CCCTTATCCTTTGTTGCC |  |
| Q*ureA*-F | TGTCTGATGTGGCGTTTA |  |
| Q*ureA*-R | GCACTTTACTGGCTTCCT |  |
| Q*ureB*-F | ATAAACCCGTGACCAAAGT |  |
| Q*ureB*-R | GAGGCGCTTGCCATAA |  |
| Q*ureC*-F | CCGATAGCCTGAATGAATG |  |
| Q*ureC*-R | TGAACTTGGCAGTACGTTAGT |  |
| Q*ureE*-F | ACGCACGGCTTCCAAC |  |
| Q*ureE*-R | CCTCAGCACCACCAAACA |  |
| Q*ureF*-F | GCCAGTTGTGACGGTATG |  |
| Q*ureF*-R | GGCGGCTTTCTTCATTT |  |
| Q*ureG*-F | AAACGGACGCTGAAAGGT |  |
| Q*ureG*-R | TGTTCATGCTCGGGTCTT |  |
| Q*ureD*-F | TGGAGGAAGGCGGTTAT |  |
| Q*ureD*-R | CGGTCGCAGTTGGATG |  |

Underlined sites Indicate restriction enzyme cutting sites added for cloning. Letters In boldface denote the annealing regions for overlap PCR.

**References:**

1. Simon R, Priefer U, Pühler A. 1983. A broad host range mobilization system for in vivo genetic engineering: transposon mutagenesis in gram negative bacteria. Bio/technology 1:784.

2. Rosqvist R, Skurnik M, Wolf-Watz H. 1988. Increased virulence of *Yersinia pseudotuberculosis* by two independent mutations. Nature 334:522-4.

3. Li C, Pan D, Li M, Wang Y, Song L, Yu D, Zuo Y, Wang K, Liu Y, Wei Z, Lu Z, Zhu L, Shen X. 2021. Aerobactin-mediated iron acquisition enhances biofilm formation, oxidative stress resistance, and virulence of *Yersinia pseudotuberculosis*. Front Microbiol 12:699913.

4. Dai Q, Xu L, Xiao L, Zhu K, Song Y, Li C, Zhu L, Shen X, Wang Y. 2018. RovM and CsrA negatively regulate urease expression in *Yersinia pseudotuberculosis*. Front Microbiol 9:348.

5. Hu Y, Lu P, Wang Y, Ding L, Atkinson S, Chen S. 2009. OmpR positively regulates urease expression to enhance acid survival of *Yersinia pseudotuberculosis*. Microbiology-Sgm 155:2522-2531.

6. Song Y, Xiao X, Li C, Wang T, Zhao R, Zhang W, Zhang L, Wang Y, Shen X. 2015. The dual transcriptional regulator RovM regulates the expression of AR3- and T6SS4-dependent acid survival systems in response to nutritional status in *Yersinia pseudotuberculosis*. Environ Microbiol 17:4631-45.
